# Effect of Fosfomycin on Cyclosporine Nephrotoxicity

**DOI:** 10.3390/antibiotics9100720

**Published:** 2020-10-21

**Authors:** Juan Antonio Ortega-Trejo, Rosalba Pérez-Villalva, José M. Arreola-Guerra, Victoria Ramírez, José Sifuentes-Osornio, Norma A Bobadilla

**Affiliations:** 1 Molecular Physiology Unit Instituto de Investigaciones Biomédicas, Unidad de Fisiología Molecular (UNAM), Vasco de Quiroga No. 15, Tlalpan 14080, Mexico; tonybaj_music@hotmail.com (J.A.O.-T.); melibiosa@hotmail.com (R.P.-V.); 2 Departments of Nephrology, Instituto Nacional de Ciencias Médicas y Nutrición Salvador Zubirán, Mexico City 14080, Mexico; 3 Departments of Medicine Instituto Nacional de Ciencias Médicas y Nutrición Salvador Zubirán, Mexico City 14080, Mexico; dr.jmag@gmail.com (J.M.A.-G.); jose.sifuenteso@incmnsz.mx (J.S.-O.); 4 Internal Medicine Department, Centenario Hospital Miguel Hidalgo, Aguascalientes 20259, Mexico; 5 Departments of Experimental surgery Instituto Nacional de Ciencias Médicas y Nutrición Salvador Zubirán, Mexico City 14080, Mexico; victoria.ramirezg@incmnsz.mx

**Keywords:** tubular injury, cytokine immunomodulation, vasoconstriction, rats, fosfomycin

## Abstract

Fosfomycin (Fos) has emerged as a potential treatment against multidrug-resistant organisms, however, there has been little work done on its influence on calcineurin inhibitor nephrotoxicity (CIN). This study was designed to evaluate the effect of Fos in combination with cyclosporine (CsA) on CIN. Two sets of experiments were undertaken. In the first, Wistar rats received different doses of Fos: 0, 62.5, 125, 250, and 500 mg/kg. In the second, rats were divided into four groups: control, CsA 15 mg/kg s.c., CsA + fosfomycin 62.5 mg/kg (CsA + LF), and CsA + Fos 500 mg/kg (CsA + HF). CsA was administrated daily for 14 days, whereas Fos administration started on the ninth day followed by two more doses, delivered 48 h apart. The administration of different Fos doses did not alter renal function. In contrast, CsA induced arteriolopathy, hypoperfusion, a reduction in the glomerular filtration rate, and downregulation of eNOS, angiotensinogen, and AT1R mRNA levels. Lower doses of Fos did not modify CIN. Instead, the CsA + HF group exhibited greater hypoperfusion, arteriolopathy, and oxidative stress, and increased mRNA levels of pro-inflammatory cytokines. This study shows that Fos administered by itself at different doses did not cause renal injury, but when it was given repeatedly at high dosages (500 mg/kg) in combination with CsA, it increased CIN through the promotion of greater oxidative stress and renal inflammation.

## 1. Introduction

Organ transplant recipients are at increased risk of infection due to immunosuppression treatment, instrumentation, end-organ damage, prolonged hospitalization, malnutrition, and other co-morbidities. Several studies have reported that about 55% to 68% of these patients present infections during their first year post-transplant [1,2]. After a renal transplant (RT), urinary tract infections (UTIs) are the most frequent complications during the first year, with an incidence ranging from 20% to 50% [3]. The emergence and spread of multidrug-resistant organisms (MDROs) are a global public health threat. The prevalence of MDRO infections in RT patients fluctuates from 8% to 46% depending on the type of infection, organism, and body region [3,4,5].

Because of this drug resistance, alternatives have been sought, and in the last 20 years, the use of fosfomycin has increased due to its effectiveness against gram-negative bacteria, especially those that produce beta-lactamases. Fosfomycin’s mechanism of action is due to the inhibition of UDP-N-acetylglucosamine enolpyruvyl transferase, which is an essential enzyme for bacterial wall synthesis [6,7]. Fosfomycin is an antimicrobial with a molecular weight of 259 kDa, and its use is only approved for the treatment of uncomplicated urinary tract infections [5]. Once fosfomycin is ingested, it dissociates into two constituent moieties, fosfomycin and tromethamine. The tromethamine is a proton acceptor used to stabilize the oral formulation of fosfomycin [8]. We recently conducted a randomized controlled trial at our hospital that showed the efficacy of fosfomycin for prophylaxis of urinary tract infections after kidney transplantations [9].

Calcineurin inhibitors (CNIs), such as cyclosporine A and tacrolimus, are very effective and widely used around the world for treating allograft rejection (mainly in kidney or liver transplants) and immune disorders [10,11]. These drugs act through the inhibition of calcineurin activity, which is necessary to activate the nuclear factor of activated T cells (NFAT) through its dephosphorylation [12,13,14]. Although CNIs remain the most effective available immunosuppressant agents, there is clinical concern related to the long-term nephrotoxicity that both cyclosporine A and tacrolimus produce [15]. The nephrotoxicity of CNIs is characterized by intense vasoconstriction of the afferent arteriole, which induces a significant reduction in the glomerular filtration rate (GFR). These alterations have been attributed to an imbalance of vasoactive mediators: increased vasoconstrictor factors (endothelin and thromboxane) and decreased vasodilator factors (nitric oxide and prostaglandin E2) [16,17,18,19].

In several experimental models, fosfomycin has been shown to protect against the nephrotoxic effects of cisplatin [20], aminoglycosides, and gentamicin [21]. Although, the mechanism of this effect is still unknown, it has been proposed that it could be mediated by intrinsic properties of fosfomycin’s structure, or due to some interaction that fosfomycin could have with the cellular membrane. Nevertheless, these protective effects are not always observed in clinical scenarios [22,23,24].

Despite the nephrotoxic effect of CNIs, most allograft recipients receive immunosuppressive treatment using these drugs [25,26]. In addition to this, kidney transplant patients are more susceptible to UTIs; unfortunately, clinicians have to face MDROs. Therefore, fosfomycin has emerged to treat these infections due to the low resistance of the microorganisms to this antibiotic, which has made it possible to successfully treat MDROs. However, little is known about the effect of fosfomycin on cyclosporine nephrotoxicity; thus, this study was designed to address this scenario.

## 2. Results

### 2.1. Effect of Fosfomycin on Renal Function

**Figure 1:**
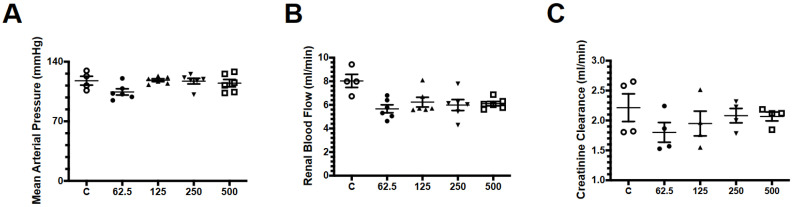
Effect of different fosfomycin doses on renal function. Five groups were studied: control (C, white circles, *n* = 4), fosfomycin 62.6 mg/kg (62.5, black circles, *n* = 6), fosfomycin 125 mg/kg (125, black triangles up, *n* = 6), fosfomycin 250 mg/kg (250, black triangles down, *n* = 6), and fosfomycin 500 mg/kg (500, white rectangles, *n* = 6). The treatments were given in three doses 48 h apart. After seven days, (**A**) mean artery pressure, (**B**) renal blood flow, and (**C**) creatinine clearance were determined.

We first analyzed whether the administration of different doses of fosfomycin could alter renal function in rats. We administered four different doses of this drug, 62.5 mg/kg (the lowest dose), 125 mg/kg (double the lowest dose), 250 mg/kg (double the previous dose), and finally 500 mg/kg (double the previous dose). In Figure 1, we show the parameters evaluated in this experiment. None of the repeated doses evaluated modified the mean arterial pressure (Figure 1A), renal blood flow (RBF) (Figure 1B), or creatinine clearance (Figure 1C). These findings allowed us to choose the lowest and highest dose to carry out this study.

### 2.2. Effect of Fosfomycin on Renal Dysfunction Induced by CsA

**Figure 2:**
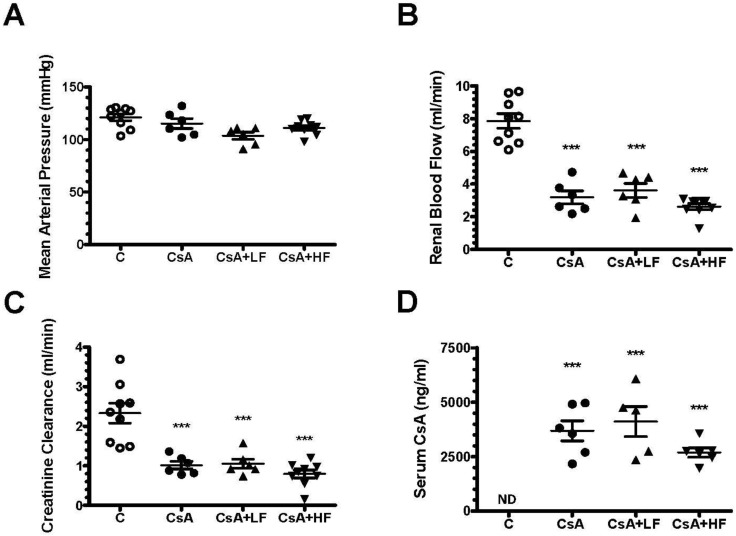
Fosfomycin does not alter renal dysfunction induced by cyclosporine. Four groups of rats were studied: control (C, white circles), cyclosporine (CsA, black circles), CsA with a low dose of fosfomycin (CsA + LF, black triangles up), and CsA with a high dose of fosfomycin (CsA + HF, black triangles down). At the end of the study, (**A**) mean artery pressure, (**B**) renal blood flow, (**C**) creatinine clearance, and (**D**) serum cyclosporine were determined. *n* = 6 to 9 per group. *** *p* < 0.001 vs. control group.

CsA administration induced nephrotoxicity, characterized by a significant reduction in renal blood flow and creatinine clearance without changes in mean arterial pressure (MAP) (Figure 2A–C). Co-administration of low or high doses of fosfomycin did not modify renal dysfunction induced by CsA (Figure 2A–C). It is important to highlight that all the experimental groups studied had similar plasma CsA levels (Figure 2D).

### 2.3. Influence of CsA and Fosfomycin on Arteriolopathy, Oxidative, and Endoplasmic Reticulum Stress

**Figure 3:**
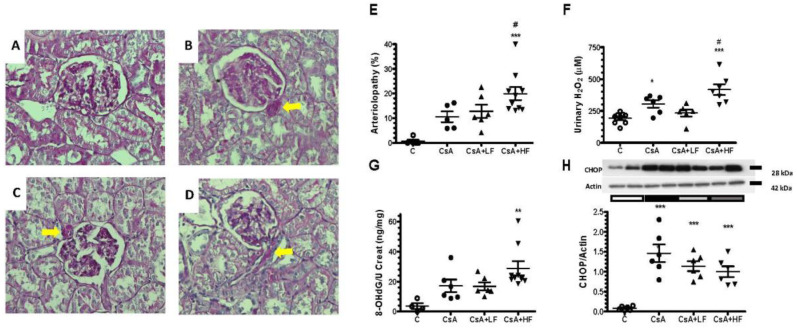
High doses of fosfomycin enhance oxidative stress and arteriolopathy induced by CsA. Representative images of periodic acid-Schiff (PAS) staining: (**A**) control, (**B**) CsA, (**C**) CsA + LF, and (**D**) CsA + HF groups (magnification: 400×). (**E**) The percentage of arteriolopathy, (**F**) urinary H_2_O_2_, (**G**) urinary 8-OHdG excretion, and (**H**) of CCAAT/enhancer-binding protein homologous protein (CHOP) expression were evaluated in control (C, white circles), cyclosporine (CsA, black circles), CsA with a low dose of fosfomycin (CsA + LF, black triangles up), and CsA with a high dose of fosfomycin treated rats (CsA + HF, black triangles down). *n* = 4 to 9 per group. * *p* < 0.05, ** *p* < 0.01, and *** *p* < 0.001 vs. control group. # *p* < 0.05 vs. CsA group.

Representative microphotographs of kidney slices periodic acid-Schiff (PAS) stained from each studied group are shown in Figure 3A–D. The characteristic arteriolopathy induced by CsA is evident in the groups receiving this drug. The corresponding quantitative analysis of the arteriolopathy percentage is depicted in Figure 3E. CsA-inducing arteriolopathy was not modified by the lower dose of fosfomycin, but arteriolopathy percentage increased significantly with the co-administration of the highest dose of fosfomycin. It has been previously reported that CsA increases mitochondrial reactive oxygen species (ROS) production and induces endoplasmic reticulum (ER) stress [27]. In this study, we confirmed greater oxidative stress in the CsA-treated group, as measured by the urinary hydrogen peroxide excretion (UH_2_O_2_V). According to our histopathological findings, the UH_2_O_2_V was not modified by the lower dose of Fos, but it was significantly increased by the co-administration of CsA with the higher dose of fosfomycin, compared to the CsA group (Figure 3F). We also evaluated oxidative DNA injury through the testing of urinary 8-OHdG levels. The urinary excretion of 8-OHdG corrected by urinary creatinine was increased in the CsA group compared to the control group (17.1 ± 4.2 vs. 3.6 ± 1.9 ng/mg of creatinine), but it did not reach statistical significance (*p* = 0.14). Similar results were found with the co-administration of the lower dose of fosfomycin. On the contrary, the urinary 8-OHdG increased by 11.3-fold in the CsA + HF group compared to the control; therefore, oxidative DNA injury was even more pronounced in this group (29.3 ± 4.1 vs. 2.6 ± 1.2 ng/mg of creatinine, as is shown in Figure 3G). Additionally, we evaluated the protein levels of CCAAT/enhancer-binding protein homologous protein (CHOP) as an endoplasmic reticulum stress marker. Renal expression of CHOP was significantly increased in all the groups receiving CsA compared to the control group, and fosfomycin co-administration did not modify this effect (Figure 3H).

### 2.4. Renal mRNA Levels of Anti-Oxidative Enzymes during CsA Treatment and Fosfomycin Co-Administration

**Figure 4:**
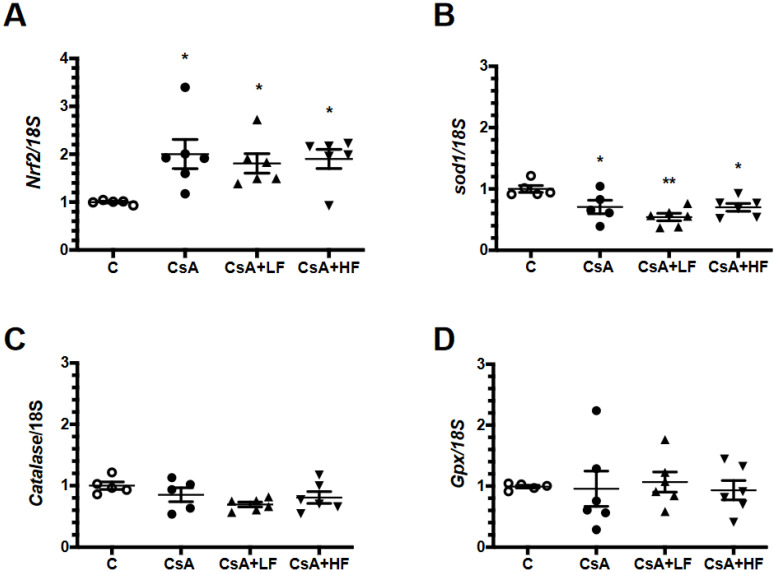
mRNA levels involved in anti-oxidative pathways. (**A**) Transcription factor *Nrf2*, (**B**) *Sod1* (**C**) *catalase*, and (**D**) *Gpx1* mRNA levels were assessed by semi-quantitative real time RT-PCR in control (C, white circles), cyclosporine (CsA, black circles), CsA with a low dose of fosfomycin (CsA + LF, black triangles up), and CsA with a high dose of fosfomycin treated rat group (CsA + HF, black triangles down)**.** *n* = 6. * *p* < 0.05 and ** *p* < 0.01 versus control group.

In order to evaluate whether a reduction in antioxidant defense mediated oxidative stress, we evaluated the mRNA levels of *Nrf2*, catalase, *Sod1*, and *Gpx1*. As shown in Figure 4A, there was a significant increase in *Nrf2* mRNA levels in the CsA-treated group, an effect that was not altered by fosfomycin co-administration. In spite of the increase in *Nrf2* transcription, *Sod1* mRNA levels were significantly decreased in all the experimental groups (Figure 4B), whereas catalase and *Gpx1* mRNA levels remained unchanged in all the groups studied (Figure 4C,D).

### 2.5. Effects of CsA and Fosfomycin on Renal Inflammation

**Figure 5:**
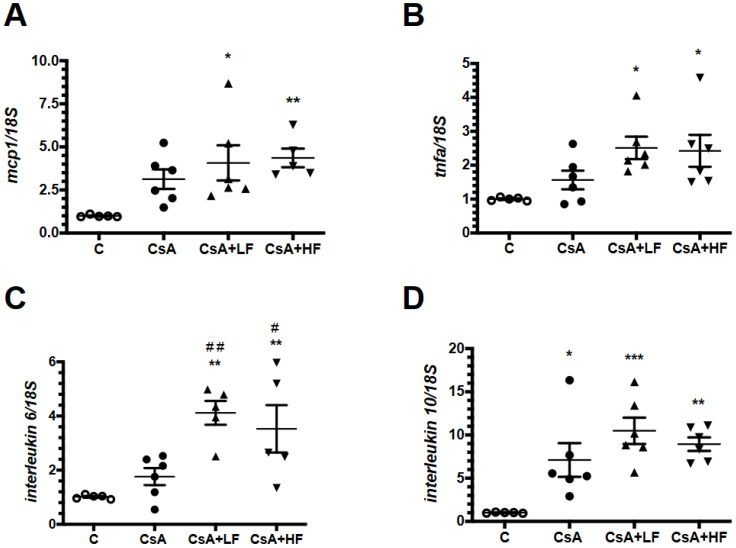
The co-administration of fosfomycin and CsA enhances the transcription of inflammatory cytokines. The mRNA levels of pro-inflammatory cytokines (**A**) *mcp-1*, (**B**) *tnf-α*. and (**C**) *IL-6*, and (**D**) *IL-10* mRNA levels in control (C, white circles), cyclosporine (CsA, black circles), CsA with a low dose of fosfomycin (CsA + LF, black triangles up), and CsA with a high dose of fosfomycin treated rats (CsA + HF, black triangles down). *n* = 6. * *p* < 0.05, ** *p* < 0.01, and *** *p* < 0.01 vs. control group. # *p* < 0.05 and ## *p* < 0.01 vs. CsA group.

Because it is known that fosfomycin could exert immunomodulation of several cytokines [28], we assessed the mRNA levels of *mcp-1, tnf-α, IL-6* and *IL-10*, as is shown in Figure 5. Although all the analyzed cytokines exhibited higher mRNA levels in the CsA-treated group, only *IL-10* reached statistical significance, and this effect was not altered by fosfomycin administration. Interestingly, the co-administration of CsA with a low or a high dose of fosfomycin induced a significant increase in *mcp-1*, *tnf-α*, and *IL-6* mRNA levels by approximately 5-, 3-, and 4-fold, respectively, when compared to the control group.

### 2.6. Renal mRNA Levels of Vasoactive Factors during CsA Treatment and Fosfomycin Co-Administration

**Figure 6:**
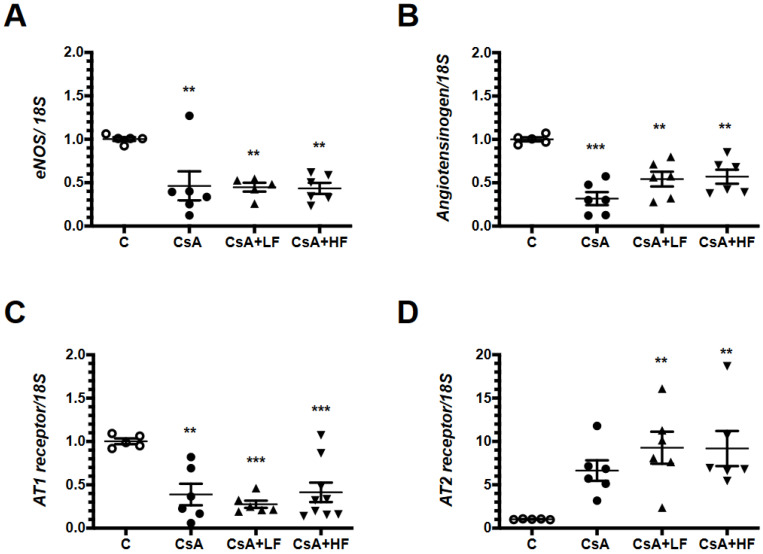
Effect of CsA and fosfomycin co-administration on vasoactive mediators. (**A**) *eNOS*, (**B**) *angiotensinogen*, (**C**) *AT1R*, and (**D**) *AT2R* mRNA levels in control (C, white circles), cyclosporine (CsA, black circles), CsA with a low dose of fosfomycin (CsA + LF, black triangles up), and CsA with high dose of fosfomycin treated rats (CsA + HF, black triangles down). *n* = 6. ** *p* < 0.01, and *** *p* < 0.01 vs. control group.

CsA nephrotoxicity is characterized by intense vasoconstriction occurring primarily in the afferent arteriole [29,30]; therefore, the behavior of vasoactive factors was evaluated. CsA administration was associated with a downregulation of *eNOS* transcription compared with the control group, and this effect was not altered by fosfomycin co-treatment (Figure 6A). In all the groups treated with CsA, the mRNA levels of *angiotensinogen* were significantly decreased (Figure 6B). *AT1 receptor* mRNA levels (that mediate vasoconstriction) were also significantly decreased in all the groups treated with CsA, and fosfomycin did not modify this behavior (Figure 6C), whereas *AT2 receptor* mRNA levels (that mediate vasodilation) were significantly increased in CsA-treated animals, but only reached statistical significance in the CsA + LF and the CsA + HF groups (Figure 6D).

### 2.7. Effects of CsA and Fosfomycin on mRNA Levels of the Endothelin Pathway

**Figure 7:**
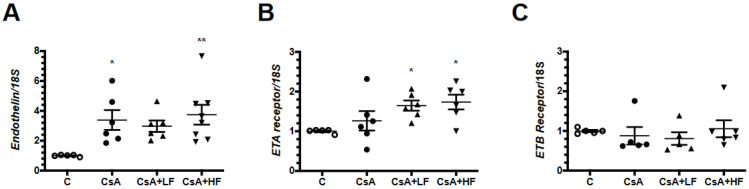
Endothelin pathway during the co-administration of CsA and fosfomycin. (**A**) *Prepro-endothelin*, (**B**) *ETA* receptor, and (**C**) *ETB* mRNA levels in control (C, white circles), cyclosporine (CsA, black circles), CsA with a low dose of fosfomycin (CsA + LF, black triangles up), and CsA with a high dose of fosfomycin treated rats (CsA + HF, black triangles down). *n* = 6. * *p* < 0.05, ** *p* < 0.01 vs. control group.

We also evaluated endothelin and its receptors. There was an increase in the *prepro-endothelin* mRNA levels in the CsA-treated groups, but only CsA and CsA + HF reached statistical significance compared to the control group (Figure 7A). Interestingly, upregulation of *ETA* mRNA levels was only seen in CsA + LF and CsA + HF groups compared to the control group (Figure 7B). The *ETB* mRNA levels were not affected by CsA, nor by fosfomycin administration (Figure 7C).

## 3. Discussion

Fosfomycin has emerged as a significant antibiotic to deal with MDROs, but little is known about its effect when it is combined with drugs that produce toxicity, as is the case of CINs. Here, we show that fosfomycin administrated in four different doses did not produce changes in the mean arterial pressure, renal blood flow, and creatinine clearance. When fosfomycin was combined with CsA, a low dosage did not modify CsA-induced renal abnormalities, but a higher dosage potentiated CsA nephrotoxicity. Notably, this adverse effect was not mediated by the fact that fosfomycin induced a higher concentration of CsA by encouraging its metabolism, since all the groups treated with this CIN had similar serum levels of CsA. While fosfomycin is excreted in the urine, almost completely unmetabolized by glomerular filtration [31], CsA is metabolized by the CYP450 family predominantly in the liver [32]. Therefore, these two drugs are not metabolized by the same route, and they have not been reported to bind to each other.

CsA nephrotoxicity was characterized by a significant decrease in RBF and GFR without changes in MAP, as we have previously reported [29,30]. Although co-administration of fosfomycin did not alter renal dysfunction more than that induced by the administration of CsA, it did produce greater structural damage only when the highest dose was administrated. So, the highest percentage of arteriolopathy was seen in the CsA + HF group.

Oxidative stress plays a crucial role in the pathogenesis of CsA nephrotoxicity. Reduced antioxidant capacity has been shown to make rats more susceptible to nephrotoxic agents [33]. Previous studies in dibekacin, vancomycin, and gentamicin nephrotoxicity showed that fosfomycin could exert beneficial effects by reducing oxidative stress [20,21,34]. However, Yanagida et al. demonstrated that the protective effects of fosfomycin are more related to the protection of the integrity of the lysosomal membrane than to a direct chelating or antioxidant action [21]. In our study, we did not observe that the lower dose of fosfomycin could have antioxidant properties. Also, we found that the higher dose induced greater oxidative stress when it was combined with CsA. Since greater UH_2_O_2_V and U8-OHdG were detected, an increase in CHOP, an endoplasmic reticulum stress marker, was observed. Remarkably, there was an increase in *Nrf2* mRNA levels in all the CsA-treated groups; *Nrf2* is a transcription factor that controls the expression of antioxidant enzymes [35], and it is very plausible that Nrf2 is produced to decrease the ROS damage induced by the CsA-hypoxic state. However, no changes were detected in mRNA levels of antioxidant enzymes, except in *Sod1*, which had a decrease. Therefore, upregulation of *Nrf2* mRNA levels were not enough to prevent ROS kidney damage caused by CsA, and even less when it was co-administered with a high dose of fosfomycin.

Another important finding of our study was the elevation of cytokines transcription during the fosfomycin treatment. This immunomodulative effect of fosfomycin has already been reported in T and B cells, but both pro-inflammatory and anti-inflammatory effects have been observed [36,37]. In this study, we showed that fosfomycin increases the pro-inflammatory cytokines *mcp-1, tnf-α*, and *IL-6* in CsA nephrotoxicity. These cytokines can promote the recruitment of immune cells, which in turn produce proteases, myeloperoxidase, and ROS. All of these produce greater vascular permeability and alter the integrity of tubular epithelial and endothelial cells, thus perpetuating kidney damage [38]. Therefore, these cytokines could be partly responsible for the increased oxidative stress found in the groups combining CsA and fosfomycin. More in-depth research needs to be done to clarify this finding.

Renal vasoconstriction induced by CsA has been attributed to an imbalance in vasoactive mediators. On the one hand, there is an increase in vasoconstrictor factors, such as thromboxane A2, angiotensin II, and endothelin, and on the other, there is a decrease in NO, a key vasodilator factor [19,29,30,39]. CsA nephrotoxicity was also associated with a significant reduction in *eNOS* and a significant increase in *prepro-endothelin* mRNA levels. These effects were not modified by fosfomycin, but it promoted higher *ETA* receptor mRNA levels in both the CsA + LF and CsA + HF groups, suggesting that fosfomycin could mediate greater vasoconstriction. However, renal micropuncture studies are needed to determine afferent and efferent arterioles resistance to confirm this finding.

The *AT1 receptor* plays an important role in the vasoconstrictive effects of angiotensin II. It has been proposed that under pathological conditions, one of the compensatory mechanisms to counteract the contractile response is the upregulation of AT2R, which mediates vasodilation [40]. Although we found an increase in *AT2 receptor* mRNA levels in the CsA + LF and CsA + HF groups, it was not enough to modify the renal vasoconstriction induced by CsA.

In summary, we show here that repeated low doses of fosfomycin do not alter cyclosporine-induced nephrotoxicity. However, CsA-induced kidney damage is increased when a much higher (8-fold) dose of fosfomycin is used. Increased arteriolopathy, oxidative stress, and proinflammatory cytokine mRNA levels evidenced this potentiation in CsA nephrotoxicity. 

## 4. Materials and Methods

### 4.1. Ethics

All experiments involving animals were conducted in accordance with the National Institute of Health’s Guide for the Care and Use of Laboratory Animals and with the Mexican Federal Regulation for animal reproduction, care and experimentation (NOM-062-ZOO-2001). The Animal Care and Use Committee at Instituto Nacional de Ciencias Médicas y Nutrición Salvador Zubirán (NMM-1401), Mexico City, approved the study.

### 4.2. Subject Inclusion, Housing, Randomization, and Size Calculation

The rats were bred in our institutional housing facility. Male Wistar rats weighing between 310 and 340 g (approximately 3 months old) were used for this study. All rats were maintained in a controlled environment with a constant temperature and humidity along with a fixed 12:12-h light-dark cycle. All rats had free access to standard rat chow and water ad libitum. All animals provided by our housing facility were in good health, and each rat was randomly assigned to a control or experimental group. The sample size calculation was performed using a difference in means analysis, assuming the presence of renal dysfunction induced by CsA that we previously demonstrated [18,31,32], and using a power level of 95% and a *p* value of 0.05. The calculated sample size was *n* = 6. During the follow-up, a humanitarian end-point guideline was maintained evaluating physical appearance, weight loss, the presence of infections, mobility, etc., and no rats merited exclusion from the study.

### 4.3. Effect of Different Doses of Fosfomycin on Renal Function

To evaluate whether fosfomycin exerted an effect by itself on renal hemodynamics, we included 28 male Wistar rats that were divided to receive different doses of fosfomycin in drinking water: 62.5, 125, 250, and 500 mg/kg (*n* = 6 for each dose). Each rat received its corresponding fosfomycin dose every 48 h until three doses were reached, and then they were studied 24 h after they received the last dose. All rats were compared to the control group (*n* = 4). Based on our results, we decided to use the highest dose (500 mg/kg) and a fourth of the highest dose (62.5 mg/kg) for the rest of the study. The lower dose of fosfomycin was chosen to simulate a clinical scenario [6], and the higher dose was based on previous studies performed in rats [20,27].

### 4.4. Effect of Fosfomycin Administration on Cyclosporine Nephrotoxicity

Twenty-eight male Wistar rats weighing between 310 and 340 g were included and randomly separated into four groups as follows: (1) control rats that received 0.1 mL of olive oil s.c. per day as a vehicle during 14 days, (2) rats that received CsA (15 mg/kg), (3) rats that received CsA with three doses of fosfomycin (62.5 mg/kg) in their drinking water, 48 h apart starting at day 9 (CsA + LF), and (4) rats that received CsA for 14 days with three doses of fosfomycin (500 mg/kg) in their drinking water separated by 48 h starting at day 9 (CsA + HF). All animals were kept in a 12:12 day-night cycle and had free access to water and food. CsA (CAS number 59865-13-3, molecular formula C_62_H_111_O_12_, purity > 98.0%, relative molecular mass 1202.61 Da) was used to induce nephrotoxicity [28]. The fosfomycin used has the CAS number: 78964-85-9, molecular formula C_7_H_18_NO_7_P, purity > 98%, and a relative molecular mass of 259.19 kDa.

### 4.5. Functional Parameters

On the 6th or the 13th day of the study, the rats were placed in metabolic cages to collect 24-h urine samples. Serum and urine creatinine concentrations were measured using the QuantiChrom creatinine assay kit (DICT-500, BioAssay System, Hayward, CA, USA) following the manufacturer’s instructions. Creatinine clearance was obtained using the standard formula: CrCl = (U × V)/P, where U is the creatinine concentration in the urine, V is the urine flow rate, and P is the plasma creatinine concentration.

At the end of the study, rats were anesthetized with sodium pentobarbital (30 mg/kg, ip) and placed on a homoeothermic table to maintain core body temperature at 37 °C. The trachea and femoral artery were cannulated with polyethylene tubing PE-240 and PE-50. The mean arterial pressure (MAP) was monitored with a pressure transducer (model p23 db, Gould, Hato Rey, PR) and recorded on a polygraph (Grass Instruments, Quincy, MA, USA). An ultrasound transit-time flow probe was placed around the left artery and filled with ultrasonic coupling gel (HR Lubricating Jelly, Carter-Wallace, New York, NY, USA) to record the renal blood flow (RBF). The left kidneys were removed and macroscopically separated into cortex and medulla; after that, they were frozen in liquid nitrogen and stored at −80 °C. The right kidneys were perfused with physiological solution and a freshly prepared 4% formalin buffer to fix the tissue.

### 4.6. Light Microscopy Analysis

After appropriate dehydration, renal tissue was embedded in paraffin, sectioned every 3 µm, and stained with periodic acid-Schiff (PAS). At least ten subcortical fields were recorded from each kidney slide using a digital camera incorporated in a Nikon Light microscope (Melville, NY, USA) (magnification 200×). In each microphotograph, the total glomeruli were counted, and the percentage of damaged glomeruli due to arteriopathy was determined.

### 4.7. Gene Expression Analysis

Total RNA was isolated from the left kidneys using the TRIzol method (Invitrogen, Carlsbad, CA, USA), and RNA integrity was confirmed by 1% agarose gel electrophoresis. Reverse transcription was carried out with 1 µg of total RNA and 200 U of Moloney murine leukemia virus reverse transcriptase (Invitrogen). The mRNA levels of AT1 receptor (Rn00569139_m1), AT2 receptor (Rn00560677_s1), IL-6 (Rn01410330_m1), IL-10 (Rn99999012_m1), endothelin (RN00561129_m1), ETA and ETB receptors (Rn00561137_m1 and Rn00569139_m1), eNOS (Rn02132634_s1), catalase (Rn00560930_m1), SOD1 (Rn00566938_m1), Gpx1 (Rn00577994_g1), AGT (Rn00593114_m1), MCP-1 (Rn00580555), and TNF-α (Rn99999017_m1) were quantified by real-time PCR on an ABI Prism 7300 sequence Detection System (TaqMan, ABI, Foster City, CA, USA). Eukaryotic 18S rRNA was used as the endogenous control. The relative expression was calculated with the comparative threshold cycle (Ct) method.

### 4.8. Urinary Hydrogen Peroxide Excretion

The amount of hydrogen peroxide (H_2_O_2_) in urine was determined with an Amplex Red Hydrogen Peroxide/Peroxidase Assay Kit (Invitrogen) according to the manufacturer’s instructions. The assay was performed with a standard curve of H_2_O_2_ 1–10 μM. A volume of 50 μL of urine or standard was placed in a microplate, 50 μL of Amplex Red reagent-horseradish peroxidase (HRP) was then added, and the samples were incubated for 30 min at room temperature and protected from light. The plate was read at 560 nm.

### 4.9. Serum Cyclosporin

Serum CsA concentration was determined using a fluorescent polarization immunoassay (FPIA). In this technique, CsA competes with a similar fluorescein-labeled molecule for antibody binding sites within a solution. The greater the amount of CsA in the sample, the lower the emission of polarized light that is detected.

### 4.10. Western Blot Analysis

Total renal proteins were isolated from the renal cortex of each rat and homogenized in a lysis buffer (50 mM HEPES pH 7.4, 250 mM NaCl, 5 mM EDTA, 0.1% NP-40) and complete protease inhibitor (Roche, Basel, SZ). Protein samples were resolved by 8.5% SDS-PAGE electrophoresis and electroblotted into polyvinylidene fluoride membranes (Millipore, Burlington, MA, USA). Membranes were then blocked with 5% blotting-grade non-fat dry milk and were then incubated in 5% blotting-grade non-fat dry milk with their respective antibodies. A specific antibody against CHOP (Cell Signaling Technology, Danvers, MA, USA, 1:2500) was used. After incubation with the primary antibody, the membranes were washed and incubated with their secondary antibody (Santa Cruz Biotechnology, Inc., Santa Cruz, CA, USA anti-mouse, 1:5000). As a loading control, the membranes were incubated overnight at 4 °C with anti-actin antibody conjugated to HRP (Abcam, Cambridge, UK, 1:1,500,000 dilution). Proteins were detected with an enhanced chemiluminescence kit (Millipore) and autoradiography, following the manufacturer’s recommendations. The bands were scanned for densitometric analysis.

### 4.11. Urinary 8-OHdG Excretion

The urinary level of 8-OHdG, as an oxidative DNA damage marker, was evaluated using a commercially available ELISA kit (JalCA, Fukuroi, Shizuoka, JP) following the manufacturer’s instructions.

### 4.12. Statistical Analysis

The results are presented as the mean ± standard error (*SE*). The significance of the differences between the groups was assessed by analysis of variance (ANOVA) using the Bonferroni post hoc test for multiple comparisons. Statistical analyses were performed using GraphPad Prism version 8.00 for Mac OS X (GraphPad Software, La Jolla, CA, USA). Statistical significance was defined as *p* value < 0.05.

## 5. Conclusions

Treatment with fosfomycin as a therapeutic alternative in patients with infections caused by multidrug resistant bacteria is increasing. The present preclinical study advises us about the potentiation of CsA toxicity when it is combined with fosfomycin; therefore, caution should be exerted when high doses of fosfomycin are used in child and adult organ transplant recipients receiving calcineurin inhibitors.
